# Investigational Treatments in Phase I and II Clinical Trials: A Systematic Review in Asthma

**DOI:** 10.3390/biomedicines10092330

**Published:** 2022-09-19

**Authors:** Luigino Calzetta, Marina Aiello, Annalisa Frizzelli, Elena Pistocchini, Beatrice Ludovica Ritondo, Paola Rogliani, Alfredo Chetta

**Affiliations:** 1 Respiratory Disease and Lung Function Unit, Department of Medicine and Surgery, University of Parma, 43126 Parma, Italy; 2 Unit of Respiratory Medicine, Department of Experimental Medicine, University of Rome “Tor Vergata”, 00133 Rome, Italy

**Keywords:** asthma, efficacy, investigational, Phase I, Phase II, RCT

## Abstract

Inhaled corticosteroids (ICS) remain the mainstay of asthma treatment, along with bronchodilators serving as control agents in combination with ICS or reliever therapy. Although current pharmacological treatments improve symptom control, health status, and the frequency and severity of exacerbations, they do not really change the natural course of asthma, including disease remission. Considering the highly heterogeneous nature of asthma, there is a strong need for innovative medications that selectively target components of the inflammatory cascade. The aim of this review was to systematically assess current investigational agents in Phase I and II randomised controlled trials (RCTs) over the last five years. Sixteen classes of novel therapeutic options were identified from 19 RCTs. Drugs belonging to different classes, such as the anti-interleukin (IL)-4Rα inhibitors, anti-IL-5 monoclonal antibodies (mAbs), anti-IL-17A mAbs, anti-thymic stromal lymphopoietin (TSLP) mAbs, epithelial sodium channel (ENaC) inhibitors, bifunctional M_3_ receptor muscarinic antagonists/β_2_-adrenoceptor agonists (MABAs), and anti-Fel d 1 mAbs, were found to be effective in the treatment of asthma, with lung function being the main assessed outcome across the RCTs. Several novel investigational molecules, particularly biologics, seem promising as future disease-modifying agents; nevertheless, further larger studies are required to confirm positive results from Phase I and II RCTs.

## 1. Introduction

The 2022 Global Initiative for Asthma (GINA) report [1] describes asthma as a heterogeneous disease, often characterised by chronic airway inflammation, with a history of respiratory symptoms, including wheeze, shortness of breath, chest tightness, and cough that vary over time and in intensity, along with variable expiratory airflow limitation.

The long-term goals of asthma management are to achieve symptom control, reduce the risk of exacerbations and mortality, preserve lung function, and minimise drug-related side effects [1]. The stepwise approach used for pharmacological treatment in asthma mandates an iterative cycle of assessment, adjustment of pharmacological and nonpharmacological treatment, and review of the therapeutic response [1].

Over the last 30 years, inhaled corticosteroids (ICS) have been the mainstay of asthma treatment, with the long-acting β_2_-adrenoceptor agonist (LABA) formoterol/ICS combination serving as the preferred controller and/or reliever therapy, depending on asthma severity [2]. Nevertheless, this therapeutic option has become increasingly unattractive due to its inability to alter the natural course of the disease, including asthma progression [3]. Although ICS are clinically efficacious in most asthmatics, a considerable subset of patients (3–10%) remain uncontrolled despite optimal therapeutic adherence and proper inhaler technique [4]. Even after using the highest dosage of ICS, such individuals do not achieve control over their symptoms, and often need to step up to treatment with oral corticosteroids (OCS) in order to avert future episodes of life-threatening exacerbations [5].

This variability in the therapeutic response is the result of the highly heterogeneous nature of asthma [6] in terms of pathogenesis, disease severity, and outcomes [7]. Asthma is nowadays referred to as an umbrella diagnosis encompassing a plethora of endotypes and clinical phenotypes that vary from mild to severe forms [8].

More recently, the management of asthma has evolved from a blockbuster approach of “one size fits all” to a more personalised one, which treats the patient rather than the disease. In the early 2000s, the introduction of biological therapies directed towards specific inflammatory pathways advanced the improvement of asthma outcomes, initially with the anti-IgE monoclonal antibody (mAb) omalizumab [9], followed 10 years later by the approval of the mAbs anti-interleukin (IL)-5 mepolizumab and reslizumab, and the anti-IL-5Rα benralizumab [10]. The newest treatment options for severe uncontrolled asthma include the mAbs anti-IL-4/IL-13 dupilumab [11] and the anti-thymic stromal lymphopoietin (TSLP) tezepelumab [12]. Such mAbs have noteworthy properties, reducing asthma exacerbations with an OCS sparing effect [10].

In recent years, a lot of effort has been put into the development of a more personalised approach [13]. The ability to target specific inflammatory mediators and cellular pathways via highly selective therapeutic agents has progressively revolutionised the treatment of a complex, heterogeneous disorder such as asthma [14]. Although current medications may improve symptom control, QoL, and the frequency and severity of exacerbations, they do not really induce asthma remission [3].

Therefore, the aim of this review was to systematically assess the investigational agents in Phase I and II under development in the last five years, in order to understand whether there is some emerging drug and/or formulation that might be developed in the future for effective treatment of asthmatic patients.

## 2. Materials and Methods

### 2.1. Review Question

The question of this systematic review was to assess whether some of the current investigational agents in Phase I and II clinical trials (CTs) might be suitable for effective treatment of asthmatic patients.

### 2.2. Search Strategy

**Figure 1:**
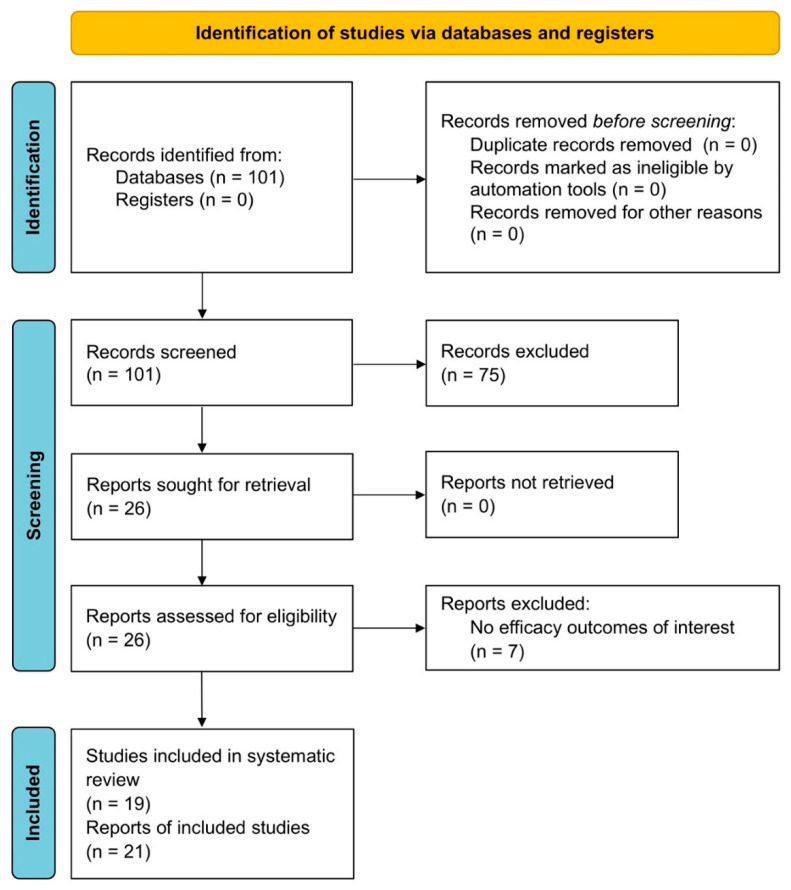
PRISMA 2020 flow diagram for the identification of the RCTs included in the qualitative and quantitative syntheses. PRISMA: Preferred Reporting Items for Systematic Reviews and Meta-Analyses; RCT: randomised controlled trial.

The protocol of this synthesis of the current literature has been registered to the international prospective register of systematic reviews (PROSPERO, Protocol ID: CRD42022336605), and performed in agreement with the Preferred Reporting Items for Systematic Reviews and Meta-Analyses Protocols (PRISMA-P) [15], with the relative flow diagram reported in Figure 1. This study satisfied all the recommended items reported by the PRISMA 2020 checklist [16].

The PICO (Patient problem, Intervention, Comparison, and Outcome) framework was applied to develop the literature search strategy and question, as previously reported [17]. Namely, the “Patient problem” included asthmatic patients; the “Intervention” regarded investigational agents in Phase I and II CTs; the “Comparison” was performed with respect to placebo (PCB) and/or active comparators; the assessed “Outcomes” were lung function, symptoms control, blood eosinophil count (BEC), fractioned exhaled nitric oxide (FENO), exacerbations and hospitalisations, the use of rescue medications, and quality of life (QoL).

A comprehensive literature search was performed for Phase I and II CTs, written in English and investigating the impact of investigational treatments in patients with asthma. The search was performed in ClinicalTrials.gov in order to provide relevant studies available within the past 5 years (from May 2017 to May 2022).

The term “asthma” was searched for the disease, “Interventional Studies (Clinical Trials)” was selected for the study type, “Terminated” and “Completed” were chosen for the recruitment status, and “Early Phase I”, “Phase I”, and “Phase II” were selected in the Additional Criteria of the Advanced Search in the ClinicalTrials.org database.

### 2.3. Study Selection

Randomised controlled trials (RCTs) reporting results concerning the efficacy profile of investigational treatments vs. PCB and/or active comparators were included in the systematic review.

Two reviewers independently checked the relevant studies identified from ClinicalTrials.gov. The studies were selected in agreement with previously mentioned criteria, and any difference in opinion regarding eligibility was resolved by consensus.

### 2.4. Data Extraction

Data from included studies were extracted and checked for study references, a NCT number identifier, study duration, treatments and comparators with doses and regimen of administration, number and characteristics of analysed patients, age, gender, smoking habit, forced expiratory volume in 1 s (FEV_1_), peak expiratory flow (PEF), Asthma Control Questionnaire (ACQ) score and other outcomes related to the impact on symptoms, BEC, FENO, asthma exacerbations, hospital admissions, rescue medication use, Asthma QoL Questionnaire (AQLQ) score, St George’s Respiratory Questionnaire (SGRQ) score, and study quality assessment via the Jadad Score [18] and Cochrane Risk of Bias 2 (RoB 2) [19].

### 2.5. Endpoints

The co-primary endpoints of this systematic review were the impact of investigational treatments on lung function and symptoms control.

The secondary endpoints were the impact of investigational treatments on blood eosinophil count, FENO, exacerbations and hospitalisations, the use of rescue medications, and QoL.

### 2.6. Strategy for Data Synthesis

Data from original papers were extracted and reported via qualitative synthesis, and the statistical significance was set at *p* < 0.05.

### 2.7. Quality Score

The summary of the risk of bias for each included randomised trial was analysed via the Jadad score [18] and Cochrane RoB 2 [19]. The weighted assessment of the overall risk of bias was analysed via the Cochrane RoB 2 [19] using the robvis visualisation software [20,21]. The Jadad score, with a scale of 1–5 (with a score of 5 being the best quality), was used to assess the quality of the papers concerning the likelihood of bias related with randomisation, double blinding, withdrawals, and dropouts [18]. Studies were considered of low quality at Jadad score < 3, of medium quality at Jadad score = 3, and of high quality at Jadad score > 3. The weighted assessment of the risk of bias was analysed via the Cochrane RoB 2 tool [19] by using the robvis visualisation software [20,21].

Two reviewers independently assessed the quality of individual studies, and any difference in opinion about the quality score was resolved by consensus.

## 3. Results

### 3.1. Study Characteristics

Of the 101 records identified in the ClinicalTrials.gov database, 75 documents were excluded due to inconsistency between the study title and the PICO framework or because no results were available. Among the remaining CTs, 19 RCTs were deemed eligible for the systematic review.

Study results for seven RCTs [22,23,24,25,26,27,28,29] were retrieved from ClinicalTrials.gov, results for five RCTs [30,31,32,33,34,35,36,37,38,39] were published in full text articles, and data for two RCTs [40,41,42,43] were obtained through the European Union (EU) Clinical Trial Register. Results for two RCTs [44,45,46,47] were available only from conference abstracts or posters, data for one RCT [48,49,50] were retrieved from both the EU Clinical Trial Register and abstract, and for another RCT, they were retrieved from both ClinicalTrials.gov and the abstract [51,52]. Results for one RCT [53,54] were provided on the pharmaceutical company’s website. The main characteristics of the studies included in the systematic review are reported in Table 1.

### 3.2. IL-4Rα Inhibitor

Antagonising the IL-4 receptor α subunit (IL-4Rα) interferes with the downstream IL-4/IL-13 signalling, which is central to the pathogenesis of asthma [55]. As a matter of fact, IL-4 regulates the proliferation and survival of T helper 2 (Th2) cells as well as immunoglobulin E (IgE) synthesis, while IL-13 is implicated as a key effector in AHR, mucus hypersecretion, ASM alterations, and subepithelial fibrosis [56].

In a Phase I RCT [46,47], mildly asthmatic patients received nebuliser treatment with the IL-4Rα inhibitor AZD1402 (PRS-060) at delivered doses of 2–60 mg twice daily (BID) to establish its efficacy profile. After a single administration, AZD1402 induced a rapid decrease in the FENO level, with a significant (*p* < 0.05) percentage reduction vs. PCB between 24.0% (95%CI 1.8–41.0) and 36.4% (95%CI 22.0–48.0) across all doses. No data are available for lung function and symptoms control [46,47].

### 3.3. Anti-IL-5 mAbs

Targeting BEC reduction through the inhibition of IL-5 represents an established therapeutic option in severe asthma [57]. Depemokimab (GSK3511294) is a subcutaneously administered anti-IL-5 mAb, designed for improved affinity and long-acting IL-5 suppression compared to the currently approved anti-IL-5 mAbs, and it has been evaluated in a first-in-human Phase I RCT [30,31] enrolling mild to moderate asthmatic patients with BEC ≥ 200 cells/μL at screening.

A single administration of depemokimab generally improved lung function parameters with an increase in the dose from 2 mg to 300 mg. Depemokimab 300 mg induced a greater improvement from baseline in FEV_1_ (240 mL (95%CI 68–412)) vs. PCB (105 mL (95%CI not calculated)) and in percent predicted normal FEV_1_ (7.65% (95%CI 1.76–13.54)) vs. PCB (3.85% (95%CI not calculated)). No data are available for symptoms control [30,31].

Across all doses, depemokimab markedly decreased the circulating BEC by >48.0% 24 h post-dose, and reductions of 54.0% and 53.0% were observed in patients treated, respectively, with depemokimab 100 mg and 300 mg. The duration of such marked suppression of BEC was dose dependent, and thus was maintained for longer with the increasing dose. Six months after the single-dose administration, depemokimab induced reductions in BEC of 31.0% (2 mg), 41.0% (10 mg), 72.0% (30 mg), 82.0% (100 mg), and 83.0% (300 mg) vs. PCB [30,31].

### 3.4. Anti-IL-17A mAbs

Increased expression of the Th17-derived cytokine IL-17A has been observed in sputum, airway tissue biopsies, and serum from asthmatic patients [58,59,60,61,62] and was positively associated with a more severe asthma phenotype [59,63,64] and neutrophilic inflammation [65]. Considering that Th17-high patients are less sensitive or even unresponsive to ICS [59,66] and that asthma progression differs from more treatable Th2 types of the disease [67], developing an effective therapy targeting Th17/IL-17A axis would overcome a major unmet need in severe asthma.

A Phase II RCT [22] investigated the subcutaneously administered anti-IL-17A mAb CJM112 300 mg when added to existing therapy in patients with inadequately controlled moderate to severe asthma, with low serum IgE and BEC. The effect of CJM112 treatment on trough FEV_1_ was not different from PCB, but a significant (*p* < 0.05) improvement was observed in the ACQ6 score (mean difference (MD) −0.22 units (80%CI −0.41–−0.04)) and the ACQ7 score (MD −0.23 units (80%CI −0.40 to −0.06)) vs. PCB. A higher proportion of patients receiving CJM112 had a decrease of ≥0.5 units in the ACQ7 score compared with PCB (71.7% vs. 52.8%) [22].

### 3.5. Anti-IL-33 mAbs

Upon cellular damage or allergen exposure, interleukin (IL)-33 is released as an alarmin by airway epithelial cells, airway smooth muscle (ASM) cells (ASMCs), and endothelium to trigger innate and adaptive immune responses [68]. In patients affected by severe asthma refractory to steroids, IL-33 activates type 2 innate lymphoid cells (ILC2s), which may promote persistent airway eosinophilia [69]. Targeted inhibition of IL-33 receptor (IL-33R) signalling may prevent downstream production of type 2 cytokines and chemokines [70].

Two RCTs [32,33,40,41] investigated the anti-IL-33 mAbs itepekimab (SAR440340/REGN3500) and etokimab (ANB020), and two other RCTs [23,24] assessed the efficacy of the anti-IL-33R mAb melrilimab (GSK3772847/CNTO7160).

A Phase II RCT [32,33] investigated the efficacy of subcutaneous itepekimab 300 mg administered alone or in combination with dupilumab 300 mg to patients with moderate to severe asthma, who progressively reduced and discontinued background therapy of inhaled corticosteroid/long-acting β2 adrenoceptor agonist (ICS/LABA) over 12 weeks. Itepekimab significantly (*p* < 0.05) improved trough FEV_1_ compared to PCB (MD 140 mL (95%CI 10–270)) and it was as effective as dupilumab, but no improvement was seen upon treatment with the combination therapy. Itepekimab did not increase post-bronchodilator FEV_1_ vs. PCB, but when combined with dupilmab, the improvement was significant (*p* < 0.05) (MD 130 mL (95%CI 10–250)) and comparable to that of dupilumab administered alone [32,33].

The percentage of patients with an event indicating a loss of asthma control was lower in the itepekimab (22.0%) and combination therapy (27.0%) groups vs. PCB (41.0%). The corresponding odds ratio (OR) for the comparison of itepekimab vs. PCB was significant (*p* < 0.05) (OR 0.42 (95%CI 0.20–0.88)) and similar to the OR for dupilumab vs. PCB; no difference was detected in the ORs for the comparison between combination therapy and PCB, itepekimab monotherapy, and dupilumab monotherapy. Itepekimab alone and combined with dupilumab significantly (*p* < 0.05) improved ACQ5 score vs. PCB (MD −0.42 units (95%CI −0.73–−0.12) and MD −0.32 units (95%CI −0.63–−0.01), respectively), and the effect was similar to that observed with dupilumab [32,33].

The BEC significantly (*p* < 0.05) decreased upon treatment with itepekimab administered alone or combined with dupilumab vs. PCB, and the effect was significantly (*p* < 0.05) different from that induced by dupilumab monotherapy, which, as expected [32], transiently induced blood eosinophilia. The FENO level was significantly (*p* < 0.05) lowered in the itepekimab group, although the magnitude of reduction was lower than that observed in the combination therapy and dupilumab groups. Patients treated with itepekimab administered alone or combined with dupilumab showed a significant (*p* < 0.05) improvement in their AQLQ score vs. PCB (MD 0.45 units (95% CI 0.14–0.77) and MD 0.43 units (95% CI 0.11–0.75), respectively), with an effect comparable to that of dupilumab [32,33].

In a Phase II RCT [40,41], a single dose of etokimab administered at 300 mg/100 mL via intravenous infusion did not improve FEV_1_ compared to PCB in severe eosinophilic asthma; no data are available for symptoms control.

The reduction in peripheral BEC following etokimab treatment was similar to that observed with PCB, and no difference was detected in FENO levels. The number of asthma exacerbations experienced by patients treated with etokimab was no different from those treated with PCB [40,41].

A Phase II RCT [23] reported that intravenously administering melrilimab 10 mg/kg to patients with moderate to severe asthma and allergic fungal airway disease for 12 weeks did not improve their FEV_1_ and ACQ5 score compared to PCB. No differences between melrilimab and PCB were observed with respect to the change from baseline in BEC, FENO level, and AQLQ score [23].

Another Phase II RCT [24] showed that melrilimab 10 mg/kg administered for 16 weeks to moderately severe asthmatic patients who gradually reduced and discontinued background therapy with fluticasone propionate/salmeterol (FP/SAL) 500/50 μg, did not improve trough FEV_1_ and morning and evening PEF vs. PCB. The reduction in ACQ5 score was similar with both melrilimab and PCB, but the percentage of patients who experienced loss of asthma control was lower in the group treated with melrilimab (67.0%) than with PCB (81.0%). No differences between the two treatment groups were observed in the percentage of night-time awakenings due to asthma symptoms requiring rescue medication use and in the daytime asthma symptom score [24].

The effect induced on BEC and FENO level was similar in the melrilimab and PCB groups. The percentage of patients with an asthma exacerbation requiring OCS and/or hospitalisation was higher with melrilimab (13.0%) than with PCB (7.0%). No differences between the two groups were observed in terms of daily use of rescue medications and SGRQ total score [24].

### 3.6. Anti-TSLP mAbs

Similar to IL-33, TSLP is mainly an epithelium-derived alarmin, which plays an upstream role in the initiation of type-2-driven immune responses [71]. In asthma, the number of cells expressing TSLP messenger ribonucleic acid (mRNA) within the airway epithelium and submucosa is markedly increased compared to healthy controls [72]. In a subset of patients with severe asthma, TSLP expression remained enhanced, independent of treatment with high-dose ICS or OCS [73]. Therefore, targeting TSLP signalling represents an intriguing therapeutic strategy in asthma [74].

In a Phase I RCT [53,54] the anti-TSLP mAb fragment ecleralimab (CSJ117) 4 mg was administered via a dry powder inhaler (DPI) for 12 weeks to patients with mild atopic asthma, who exhibited an early asthmatic response (EAR) and late asthmatic response (LAR) to a common inhaled allergen. Ecleralimab did not induce an attenuation in the EAR, as documented by the maximum percentage fall in FEV_1_ or as time-adjusted area under the curve (AUC), and numerically increased the minimum of the absolute in FEV_1_ compared to PCB. During the LAR, ecleralimab significantly (*p* < 0.05) reduced the maximum percentage decrease in FEV_1_ (MD −8.42% (90%CI −15.66–−1.18)) from pre-allergen inhalation challenge and the time-adjusted AUC fall in FEV_1_ (MD −7.18% (90%CI −11.92–−2.44)), compared to PCB. Patients in the ecleralimab group showed a strong trend towards a significant (*p* = 0.05) increase in the minimum absolute FEV_1_ during LAR vs. PCB (MD 0.27% (90%CI 0.00–0.55)) [53,54]. No data are available for symptoms control [53,54].

### 3.7. LABAs

The latest GINA report recommends treating patients with inadequately controlled asthma with a triple combination of indacaterol acetate/glycopyrronium bromide/mometasone [1]. Several studies provided evidence that indacaterol maleate is potent and safe in asthmatic patients [75,76,77,78].

A Phase I RCT [34,35] compared the efficacy of the maleate salt with the acetate salt of indacaterol 150 μg vs. PCB in patients with asthma. Indacaterol maleate significantly (*p* < 0.001) improved trough FEV_1_ of 186.0 mL (95%CI 129.0–243.0), FEV_1_ AUC0-4h by 248.0 mL (95%CI 186.0–310.0), and PEF of 33.0 L/min (95%CI 25.6–40.3) vs. PCB, and it was as effective as indacaterol acetate. No data are available for symptoms control. Rescue medication use was significantly (*p* < 0.01) reduced with both indacaterol salts of 0.42 puffs/day vs. PCB [34,35].

### 3.8. SGRMs

Compared to conventional glucocorticoids, nonsteroidal, selective glucocorticoid receptor modulators (SGRM) preferentially favour transrepression over transactivation [79]. SGRM are designed to activate the GC receptor and suppress inflammation by inhibiting nuclear factor-kappa B (NF-kB) and activator protein 1 (AP-1), whilst inducing less GC response element (GRE)-driven adverse effects [80].

Phase IIb GRANIT RCT [48,49,50] enrolled patients with inadequately controlled asthma on low-dose BUD to orally receive the SGRM velsecorat (AZD7594) 50–720 μg vs. PCB or open-label fluticasone furoate (FF) 100 μg over 12 weeks. Velsecorat dose-dependently improved trough FEV_1_ over the entire treatment period. When administered at doses of 320 μg and 720 μg, velsecorat induced a trend towards a significant improvement in trough FEV_1_ compared to PCB, which was numerically lower compared to the effect of FF vs. PCB. Velsecorat 180–720 μg significantly (*p* < 0.05) improved morning PEF vs. PCB from 9.12 L/min (95%CI 0.20–18.05) to 16.60 L/min (95%CI 8.03–25.17)). Evening PEF was significantly (*p* < 0.05) increased with velsecorat 360 μg and 720 μg vs. PCB, respectively, by 10.26 L/min (95%CI 1.46–19.06) and 11.99 L/min (95%CI 3.57–20.42). The effect of velsecorat on PEF was comparable to that induced by FF vs. PCB [48,49,50].

Velsecorat administered at doses 90–720 μg significantly (*p* < 0.05) improved the ACQ5 score vs. PCB, by inducing a reduction between −0.19 units (95%CI −0.37–−0.02) and −0.27 units (95%CI −0.43–−0.10), and it was as effective as FF vs. PCB. Velsecorat 50 μg and 180–720 μg significantly (*p* < 0.05) reduced the daily asthma symptom score between −0.14 units (95%CI −0.26 ¬– −0.02) and −0.23 units (95%CI −0.35–−0.11) and improved the percentage of symptom-free days between 8.61% (95%CI 0.30–16.91) and 11.34% (95%CI 2.77–19.91) vs. PCB, to a similar extent as FF. The percentage of asthma control days significantly (*p* < 0.05) increased with velsecorat 50 μg, 360 μg, and 720 μg over the treatment period between 8.62% (95%CI 0.49–16.75) and 10.07% (95%CI 1.46–18.67), similar to FF [48,49,50].

At doses 50–180 μg, the effect of velsecorat on FENO values was not different to PCB, but when administered at 360 μg and 720 μg, the improvement was significant (*p* < 0.05) vs. PCB (MD 0.81 ppb (95%CI 0.69–0.95) and 0.65 ppb (95%CI 0.56–0.76), respectively), and comparable to that induced by FF vs. PCB [48,49,50].

Only velsecorat 360 μg significantly (*p* < 0.05) increased the percentage of rescue-free days by 11.79% (95%CI 1.49–22.09) vs. PCB, an effect that was superior to that of FF vs. PCB. Rescue medication use was significantly (*p* < 0.05) lowered with velsecorat 50 μg, 360 μg, and 720 μg vs. PCB (MD between −0.24 puffs (95%CI −0.43–−0.05) and −0.31 puffs (95%CI −0.49–−0.13), an effect similar to that induced by FF vs. PCB [48,49,50].

Velsecorat 50–720 μg significantly (*p* < 0.05) delayed the time to recurrent CompEx event (a composite endpoint combining severe asthma exacerbations and diary events) vs. PCB (hazard ratio (HR) between 0.20 (95%CI 0.100.38) and 0.58 (95%CI 0.26–0.95)). When administered at doses of 50 μg, 180 μg, 360 μg, and 720 μg, velsecorat significantly (*p* < 0.05) reduced the annualised CompEx event rate vs. PCB (MD between 0.11 (95%CI 0.04–0.25) and 0.44 (95%CI 0.20–0.94)), while at 90 μg, velsecorat induced a strong trend towards a significant reduction in the rate vs. PCB. Overall, no comparative analysis has been performed in the study between velsecorat and FF [48,49,50].

### 3.9. MABAs

Bifunctional M_3_ receptor muscarinic antagonists/β_2_-adrenoceptor agonists (MABAs) are dimeric molecules that simultaneously block M_3_ muscarinic receptors while activating β2 receptors, and thus may be readily co-formulated with anti-inflammatory agents [81,82], simplifying dosing schedules and improving patient adherence to medication. A Phase I/II RCT [42,43] reported that in asthmatic patients, the inhaled MABA CHF6366 significantly (*p* < 0.05) improved the change from pre-dose in FEV_1_ on day 1 when administered at 160 μg, but not at 40 μg, 80 μg, and 240 μg, compared to the effect induced by PCB, while no difference was detected in the change from pre-dose in FEV_1_ on day 7; no data are available for symptoms control [42,43].

### 3.10. DP_2_ Antagonists

Evidence suggests that preventing the activation of the prostaglandin D2 receptor (DP2) pathway improves symptoms of asthma and pulmonary function, and impairs any change in eosinophil shape, while indirectly inducing a reduction in the number of exacerbations in severe asthmatic patients [83].

The LEDA Phase IIb RCT [36,37] demonstrated that the DP2 antagonist GB001 given orally at 20 mg, 40 mg, and 60 mg, in addition to the standard of care therapy, induced an effect on FEV_1_, PEF, and ACQ5 that was comparable to PCB in moderate to severe asthmatic patients with a BEC of ≥250 cells/μL. Across all doses, GB001 numerically reduced the odds of asthma worsening vs. PCB, with no dose–response effect; subgroup analysis based on baseline BEC and/or FENO did not indicate greater treatment efficacy with higher values. GB001 20 mg and 60 mg induced a significant (*p* < 0.05) delay in the time to first asthma worsening compared to PCB (HR 0.72 (95% CI 0.52–0.995) and HR 0.70 (95% CI, 0.51–0.97), respectively), while GB001 40 mg induced a numerical delay vs. PCB. Treatment with GB001 20 mg, 40 mg, and 60 mg significantly (*p* < 0.05) reduced the annualised rate of asthma worsening vs. PCB (RR 0.56 (95% CI 0.39–0.80), RR 0.65 (95% CI 0.46–0.93), and RR 0.68 (95% CI 0.48–0.96), respectively) [36,37].

There was a numerical reduction in the annualised rate of severe asthma exacerbations compared to PCB [36,37].

### 3.11. Selective BTK Inhibitors

Bruton’s tyrosine kinase (BTK) is a member of the Tec family of tyrosine kinases involved in the high-affinity receptor for IgE (FcεRI)-dependent mast cell production of cytokines and degranulation [84,85], and in the IgE-mediated activation of human basophils [86]. BTK inhibitors could be useful to treat pathological mast cell responses of asthma [87].

A Phase II RCT [25] reported that orally administering remibrutinib (LOU064) 100 mg to inadequately controlled asthmatic patients did not induce an improvement in trough FEV_1_ and in morning and evening PEF compared to PCB. Changes in the ACQ5 score, in the asthma symptom score, and in the number of puffs of SABA taken daily were not different between remibrutinib and PCB groups [25].

### 3.12. ENaC Inhibitors

An imbalance in ion transport across the airway epithelium has been implicated in asthma pathogenesis. Dysfunctions in the cystic fibrosis transmembrane conductance regulator and epithelial sodium channel (ENaC) cause changes in the airway surface liquid permeation, leading to modifications of mucus rheological properties and impairment. Blocking ENaC may reduce airway water reabsorption and increase mucus moist, therefore it is considered a potential target for the treatment of asthma [88].

A Phase I RCT [26] investigated the ENaC inhibitor BI 443651 100 μg, 400 μg, and 1200 μg administered via soft mist inhaler (SMI) to patients with mild asthma following a bolus methacholine (MCh) challenge. In the single-blind, double-dummy Part 1 of the RCT, no difference was detected between BI 443651 and PCB in terms of absolute change from baseline in maximum FEV_1_ reduction. In the double-blind, double-dummy Part 2 of the RCT, only BI 443651 administered at 1200 μg significantly (*p* < 0.05) improved the maximum FEV_1_ reduction vs. PCB (MD −157 mL (90%CI −266–−47)). No data are available for symptoms control [26].

### 3.13. Pan-JAK Inhibitors

According to in vitro studies performed on inflammatory cells isolated from asthmatic patients, pan-JAK inhibitors reduced cytokine levels and showed an additive effect on lymphocyte inhibition when combined with ICS [89]. Lung inflammation was improved upon treatment with pan-JAK inhibitors in animal models of airway inflammation [90,91,92].

A Phase II RCT [27] reported that the pan-JAK inhibitor TD-8236 administered at 150 μg and 1500 μg via DPI did not improve the FEV_1_ AUC from 3 to 8 h and the maximum percentage decline in FEV_1_ from 3 to 8 h following inhaled allergen challenge compared to PCB. No data are available for symptoms control [27].

### 3.14. Anti-Fel d 1 mAbs

The secretoglobulin Fel d 1 is the major cat allergen, eliciting IgE-mediated allergic symptoms in up to 95% of individuals with a cat allergy [93,94], such as sneezing, runny nose, nasal obstruction, conjunctivitis, and/or asthma [95]. REGN1908-1909 is an anti-Fel d 1 cocktail of two IgG4 mAbs, REGN1908 and REGN1909, with a high affinity for and noncompetitive binding to distinct epitopes of Fel d 1, which prevents the allergen cross-linking of IgE-FcεRI complexes on mast cells and basophils and the consequent degranulation and release of inflammatory mediators [96,97].

A Phase II RCT [51,52] investigated whether a single dose of the subcutaneously administered REGN1908-1909 600 mg effectively reduced bronchoconstriction in mild asthmatic patients with a cat allergy for up to 3 months following cat-allergen exposure. REGN1908-1909 significantly (*p* < 0.05) increased the median time to EAR (defined as the time leading to a ≥20% reduction in FEV_1_) vs. PCB on day 8 (HR 0.36 (95%CI 0.17–0.77)), day 29 (HR 0.24 (95%CI 0.12–0.48)), day 57 (HR 0.45 (95%CI 0.22–0.89)), and day 85 (HR 0.27 (95%CI 0.13–0.56)). REGN1908-1909 significantly (*p* < 0.05) improved FEV_1_ AUC from 0 to 2 h vs. PCB at day 8 (MD 13.56% (95%CI 6.35–20.77)), at day 29 (MD 16.21% (95%CI 6.18–26.24)), at day 57 (MD 12.30% (95%CI 2.40–22.20)), and day 85 (MD 12.54% (95%CI 3.43–21.65)). No data are available for symptoms control [51,52].

### 3.15. Synthetic Amino-Benzothiazoles

The synthetic amino-benzothiazole dexpramipexole was first developed as a treatment for amyotrophic lateral sclerosis (ALS) and during the development program, a marked targeted depletion of BEC was observed in ALS patients; therefore, dexpramipexole holds promise for asthma and eosinophil-associated diseases [98].

In the EXHALE Phase II RCT [28,29], dexpramipexole (KNS-760704) orally administered at 37.5 mg, 75 mg, and 150 mg BID for 12 weeks was investigated in patients with poorly controlled moderate to severe eosinophilic asthma with an absolute BEC of ≥300 cells/μL. No differences were observed between dexpramipexole 37.5 mg and 75 mg and PCB in trough FEV_1_ and post-bronchodilator FEV_1_, while dexpramipexole 150 mg showed a numerical improvement in both outcomes vs. PCB at the end of the treatment period, and a significant increase in trough FEV_1_ at weeks 16/18 vs. PCB. The effect of treatment on the ACQ6 score was similar to that observed in the PCB group [28,29].

Dexpramipexole 37.5 mg, 75 mg, and 150 mg significantly (*p* < 0.05) reduced BEC vs. PCB (ratio to PCB of 0.45 (95%CI 0.23–0.87), 0.34 (95%CI 0.18–0.65), and 0.23 (95%CI 0.120.43), respectively). The FENO level numerically reduced upon treatment with dexpramipexole vs. PCB across all doses. No differences were observed between the treatment and PCB groups in terms of a change in AQLQ score [28,29]

### 3.16. Antifungal Triazoles

Respiratory fungal infections complicate lung diseases and, particularly in severe asthma, up to 70.0% of patients are sensitised to at least one fungal allergen [5,99,100]. In a Phase I RCT [38,39], a single dose of inhaled PC945 5 mg did not induce a change in FEV_1_ (defined as >15.0% change from baseline, measured 10 min after receiving PCB) in mild asthmatic patients, and no acute bronchospasm was observed.

### 3.17. Probiotics

Probiotics exhibited anti-inflammatory properties to modulate immune functions and were characterised by good tolerance and safety [44]. According to preliminary results of a Phase II/III RCT [44,45] in severe uncontrolled asthma, a change from baseline in ACQ score was similar in patients receiving the orally administered Probiotical^®^ and PCB; no data are available on lung function. A significant (*p* < 0.05) reduction in the percentage of sputum eosinophils was observed between baseline and after 3 months of therapy in the Probiotical^®^ group (0.5% (95%CI 0.0–2.3) vs. 0.1% (95%CI 0.0–0.5)) compared to the PCB group (4.5% (95%CI 1.5–9.3) vs. 2.4% (95%CI 1.2–9.4)) [44,45].

### 3.18. Risk of Bias

**Figure 2:**
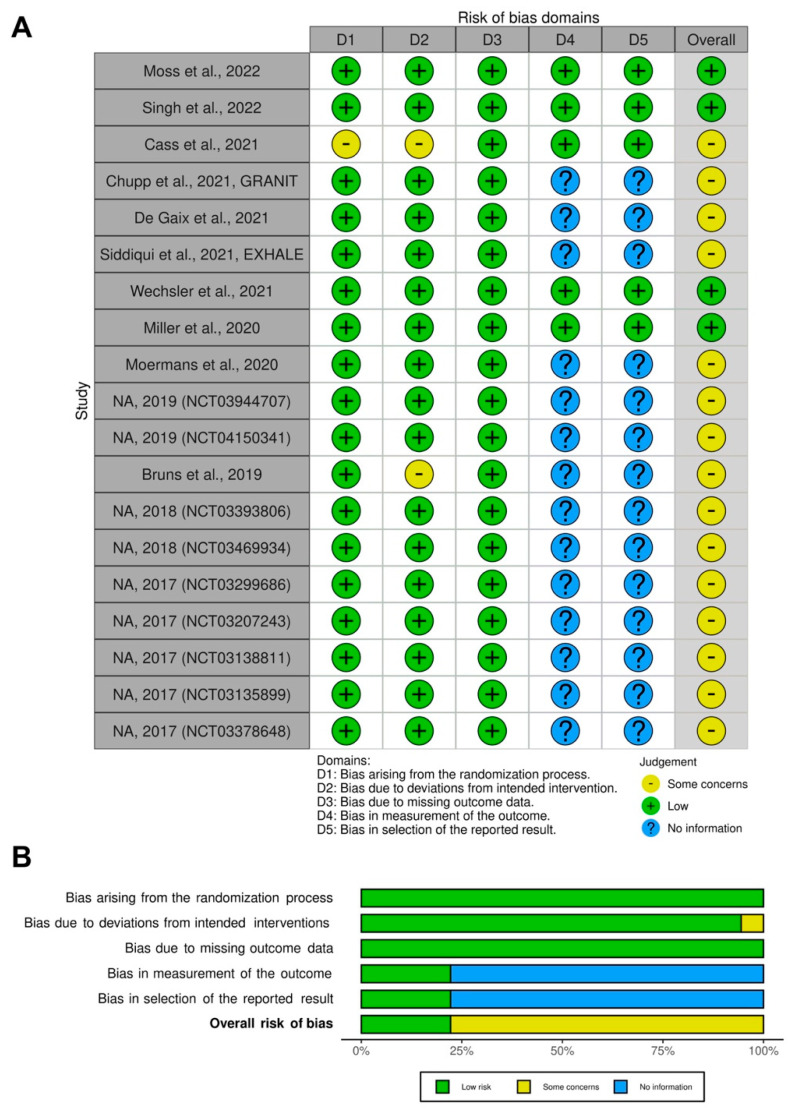
Assessment of the risk of bias via the Cochrane RoB 2 tool displayed by means of a traffic light plot of the risk of bias of the included RCTs (**A**), and weighted plot for the distribution of the overall risk of bias within each bias domain via the Cochrane RoB 2 tool (**B**) (n = 18 RCTs). Traffic light plot reports five risk of bias domains: D1, bias arising from the randomisation process; D2, bias due to deviations from intended intervention; D3, bias due to missing outcome data; D4, bias in measurement of the outcome; D5, bias in selection of the reported result. Yellow circle indicates some concerns on the risk of bias, green circle represents low risk of bias, and blue circle indicates no information. NA: not available; RCT: randomised controlled trial; RoB: risk of bias.

The traffic light plot for the assessment of each included RCT is reported in Figure 2A, and the weighted plot for the assessment of the overall risk of bias by domains is shown in Figure 2B.

All of the included RCTs (100.0%) had a low risk of bias in missing outcome data. For 18 RCTs (94.7%), there was a low risk of bias for the randomisation process, and for 17 RCTs (89.5%), the bias due to deviations from intended intervention was low. For two RCTs (10.5%), there were some concerns in the domain of bias due to deviations from intended intervention, and for one RCT (5.3%) there were some concerns for the randomisation process.

For 14 RCTs (73.7%), no information was available with regard to the risk of bias in the measurement of the outcome and selection of the reported results, as no full text articles concerning the studies have been published yet.

## 4. Discussion

An investigational medication is defined as a drug and/or formulation that has been approved for clinical testing by either the U.S. Food and Drug Administration (FDA) or the European Medicines Agency (EMA), but has not gained marketing authorisation yet [101,102]. Over the last five years, results from 19 Phase I and II RCTs on investigational agents for the treatment of asthma reported data from sixteen classes of investigational agents. Specifically, these investigational drugs included AZD1402, BI 443651, CHF6366, CJM112, depemokimab, dexpramipexole, ecleralimab, etokimab, GB001, itepekimab, melrilimab, PC945, REGN1908-1909, remibrutinib, TD-8236, velsecorat, indacaterol acetate, and a probiotic. Overall, the quality of the studies was good, although often data were not published in full text articles; thus, scarce information was available to adequately perform the RoB assessment.

The investigational anti-IL-4Rα inhibitor AZD1402, the anti-IL-5 mAb depemokimab, the anti-IL-17A mAb CJM112, the anti-TSLP mAb ecleralimab, the ENaC inhibitor BI 443651, the MABA CHF6366, and the anti-Fel d 1 mAb REGN1908-1909 were proven effective in the treatment of asthma, although data almost exclusively regarded the assessment of lung function, and thus did not allow conclusions regarding symptoms control and the secondary endpoints of this systematic review. The effectiveness of the LABA indacaterol was confirmed even when delivered using the formulation with maleate salt, which demonstrated an effect that was comparable to the currently marketed indacaterol acetate on FEV_1_, PEF, and rescue medication use reduction. Among the investigational anti-IL-33 mAbs, only itepekimab, but not etokimab and melrilimab, effectively improved asthma outcomes compared to PCB, but generally there was no further improvement observed when itepekimab was combined with dupilumab. Treatment with the SGRM velsecorat was generally superior to PCB when administered at higher doses.

Overall, investigational agents did not show superiority to active controls, with the exception of itepekimab, which significantly reduced BEC compared to dupilumab monotherapy, and velsecorat, which induced a significantly greater improvement in FEV_1_ vs. PCB compared to that produced by FF vs. PCB.

The main efficacy outcome assessed by the RCTs included in this systematic review was FEV_1_. In this respect, BI 443651, depemokimab, ecleralimab, indacaterol maleate, itepekimab, REGN1908-1909, and velsecorat produced a statistically significant improvement in lung function compared to PCB, thus representing promising add-on therapies for asthma in the future. It is also worth mentioning the synthetic amino-benzothiazole dexpramipexole, which was found to markedly reduce BEC across all the administered doses in patients with moderate to severe eosinophilic asthma, despite no significant improvement in lung function, relative to PCB [28,29].

The anti-IL-33 mAbs etokimab and melrilimab, the DP2 antagonist GB001, the selective BTK inhibitor remibrutinib, the pan-JAK inhibitor TD-8236, and the antifungal triazole PC945 induced an effect on lung function that was similar to PCB.

Although FEV_1_ is generally recognised by the research community and regulatory agencies to be a suitable variable for airflow obstruction assessment [103], it is not the most relevant endpoint for testing investigational anti-inflammatory agents, including the DP2 antagonist GB001 and the pan-JAK inhibitor TD-8236, particularly for short-term assessment. Thus, for such treatments, other efficacy endpoints should be considered in future studies. Although probiotics utilised in dietary supplements reside in a sub-category under the general umbrella term of “foods” rather than drugs, according to both the FDA [104] and the European Food Safety Authority (EFSA) [105], the probiotic Probiotical^®^ investigated in a Phase II RCT (NCT03341403) [44,45] for uncontrolled severe asthma was included in this systematic review and was treated as investigational agent. The hypothesis of such RCT [44,45] was that Probiotical^®^ could have an impact in asthmatic patients who were not optimally controlled, reducing the local and systemic inflammatory state and then improving QoL and asthma control [44,45]. This hypothesis was also supported by the evidence that certain probiotic strains have anti-inflammatory and immunomodulatory effects in pre-clinical models of asthma [106,107] and RCTs of adult asthma [108,109]. Interestingly, although dietary supplements are not subjected to the pre-market approval requirement for drugs, an investigational new drug application must be submitted to the FDA if the clinical investigation is intended to evaluate whether a dietary supplement is useful in diagnosing, curing, mitigating, treating, or preventing a disease, under the Code of Federal Regulations Part 312 [110]. In contrast, in the EU, there is still no specific regulation covering probiotics, pre-biotics, synbiotics, or postbiotics, but as suggested by The International Scientific Association of Probiotics and Prebiotics consensus statement, the definition of such products requires a health benefit; thus, it is expected that the use of any of these terms would require a health claim approval [111].

In any case, the daily administration of Probiotical^®^ showed some improvement in sputum eosinophil count after 3 months of therapy, but in agreement with the current scientific evidence, the use of probiotics as adjuvant therapy for asthma is not yet conclusive [112]. Three meta-analyses carried out to explore the potential effects of probiotics in preventing allergic diseases and asthma led to conflicting outcomes due to a high degree of heterogeneity among the studies, mostly concerning the design, the characteristics of included patients, the analysed variables, and the used probiotic strains [113,114,115].

The assessment of efficacy outcomes reported by the RCTs included in this systematic review indicates that not only were some of the investigational agents superior to PCB from a statistical point of view, but they also elicited clinically relevant effects compared to PCB in asthmatic patients, as reported in Table 2. As a matter of fact, itepekimab 300 mg Q2W, indacaterol maleate 150 μg QD, and velsecorat 720 μg QD overcame the Minimal Clinical Important Difference (MCID) [103] threshold for trough FEV_1_ or risk of asthma exacerbation. Interestingly, velsecorat 720 μg QD, itepekimab 300 mg Q2W, and itepekimab 300 mg Q2W + dupilumab 300 mg Q2W were borderline to reach the MCID threshold for ACQ or AQLQ. Indeed, these promising results need to be confirmed by Phase III studies.

A main limitation of this systematic review is that most of the included studies (11 RCT, 57.9%) had a registry record on ClinicalTrials.gov and/or EU Clinical Trial Register but no associated publication, and thus, sponsors and principal investigators are exclusively responsible for the scientific accuracy of the provided results, which may be inconsistent across all the provided studies.

Additionally, findings for three RCT of the included studies were retrieved from grey literature which were not formally and rigorously peer reviewed, and thus should be carefully interpreted due to potential publication bias [116].

There is a strong pharmacological need to look beyond current therapeutic strategies and consider further promising biological drugs for asthma that are under development and for which results have not been posted on clinical trial registries and are not available in current literature.

Asthma remission is a complex condition that can be clinically defined as a sustained absence of symptoms, optimisation or stabilisation of lung function, and no use of OCS for exacerbation treatment [117], but controversy remains regarding the threshold of each item used to assess the asthma remission itself [118]. Although these terms do not necessarily imply the absence of airway pathology, a recent point of view suggested that asthma remission may be an achievable goal, at least in asthmatic patients with the T2 phenotype [117].

In conclusion, novel investigational agents, such as biologics, may have the potential to promote disease modification. Clearly, further larger studies are needed to confirm positive results from Phase I and II RCTs. So far, most of the investigated therapies have been evaluated as add-on options to current treatment, but it would be extremely advantageous for new therapies to be effective enough to replace current pharmaceutical options in order to simplify regimens of administration.

## Figures and Tables

**Table 1:** Characteristics of the studies included in the systematic review.

Study and Year	Class of Drug	ClinicalTrials.gov Identifier and/or Company ID	Study Characteristics	Treatment Duration (wks)	Number of Analysed Patients	Drugs, Doses, and Regimen of Administration	Comparator	Route of Administration	Inhaler Device (Brand)	Patients’ Characteristics	Age (Years)	Male (%)	Current Smokers (%)	Post Bronchodilator FEV_1_ (% Predicted)	Investigated Outcome	Jadad Score
Moss et al., 2022, LEDA [36,37]	DP_2_ antagonist	NCT03683576	Phase IIb, multicentre, randomised, PCB-controlled, double-blind parallel-group study	24	481	Standard of care treatment + GB001 20 mg, 40 mg, 60 mg QD	Standard of care treatment + PCB	GB001 and PCB: PO	NA	Moderate to severe eosinophilic asthma (pre-bronchodilator FEV_1_ ≤ 85% predicted and airway reversibility or AHR; peripheral blood eosinophil count ≥ 250 cells/μL)	51.8	35.8	0.0	NA	FEV_1_, PEF, ACQ, symptoms control, and exacerbations	3
Singh et al., 2022 [30,31]	Anti-IL-5 mAb	NCT03287310	Phase I, multicentre, randomised, PCB-controlled, double-blind parallel-group study	1 day	48	As-needed SABA and stable low to moderate dose of ICS or ICS/LABA + single dose of depemokimab (GSK3511294) 2 mg, 10 mg, 30 mg, 100, 300 mg	As-needed SABA and stable low-to-moderate dose of ICS or stable low-to-moderate dose of ICS/LABA + PCB	SABA, ICS/LABA, ICS: oral inhalation; depemokimab: SC	NA	Mild to moderate asthma (pre-bronchodilator FEV_1_≥ 60% predicted, ACT score >19, and blood eosinophil count of ≥200 cells/μL)	44.0	95.8	0.0	81.0	FEV_1_ and eosinophil count	5
Cass et al., 2021 [38,39]	Antifungal triazole	NCT02715570	Phase I, single centre, two-part randomised, PCB-controlled, single-blind crossover study	1 day	9	Single dose of PC945 5 mg	PCB	Oral inhalation	NA	Mild asthma	37.7	66.7	NA	NA	FEV_1_	2
Chupp et al., 2021, GRANIT [48,49,50]	SGRM	NCT03622112	Phase IIb, multicentre, randomised, PCB-controlled, double-blind parallel-group study	12	805	Velsecorat (AZD7594) 50μg, 90 µg, 180 µg, 360 µg, 720 µg QD	FF (100 µg QD); PCB	Oral inhalation	DPI (NA)	Asthma (patients who remain symptomatic on low dose BUD [200 μg BID in Europe and 180 μg BID in US]	53.2	42.0	NA	NA	FEV_1_, PEF, ACQ, symptoms control, F_E_NO, rescue medication use, and exacerbations	3
De Gaix et al., 2021 [51,52]	Anti-Fel d 1 mAb cocktail	NCT03838731	Phase II, single-centre, randomised, PCB-controlled, double-blind parallel-group study	1 day	56	Single dose of REGN1908-1909 600 mg	PCB	SC	NA	Mild asthma with cat allergy	29.3	37.5	NA	NA	FEV_1_	3
Siddiqui et al., 2021, EXHALE [28,29]	Synthetic aminobenzothiazole	NCT04046939	Phase II, multicentre, randomised, PCB-controlled, double-blind parallel-group study	12	103	Dexpramipexole (KNS-760704) 37.5 mg, 75 mg, 150 mg BID	PCB	PO	NA	Moderate to severe eosinophilic asthma (FEV_1_ < 80% predicted and bronchodilator FEV_1_ reversibility ≥ 12% and ≥200 mL)	45.3	47.6	0.0	NA	FEV_1_, ACQ, eosinophil count, F_E_NO, and AQLQ	3
Wechsler et al., 2021 [32,33]	Anti-IL-33 mAb	NCT03387852	Phase II, multicentre, randomised, PCB-controlled, double-blind parallel-group study	12	296	Progressive withdrawal of background medication with medium-to-high-dose FP/LABA + itepekimab (SAR440340/REGN3500) 300 mg Q2W with or without dupilumab 300 mg Q2W	Progressive withdrawal of background medication with medium-to-high-dose FP/LABA + PCB or dupilumab	FP/LABA: oral inhalation; itepekimab and dupilumab: SC	NA	Moderate to severe asthma (pre-bronchodilator FEV_1_ ≥ 50% and ≤85% predicted and bronchodilator FEV_1_ reversibility ≥ 12% and ≥200 mL; ≥1 severe exacerbation within 12 months prior to screening; FEV_1_ ≥ 20% reduction in response to a provocative concentration of inhaled methacholine of <8 mg/mL within 12 months prior to screening)	49.1	36.0	0.0	NA	FEV_1_, ACQ, symptoms control, eosinophil count, and F_E_NO	3
Miller et al., 2020 [34,35]	LABA	NCT03257995	Phase II, multicentre, three-period complete block, randomised, PCB-controlled, double-blind crossover study	2	54	Background ICS medication and SABA + indacaterol maleate 150 μg QD	Background ICS medication and SABA + indacaterol acetate 150 μg QD; PCB	Oral inhalation	DPI (Breezhaler)	Asthma (pre-bronchodilator FEV_1_ ≥ 50% and ≤90% predicted normal, increase in FEV_1_ ≥ 12% and ≥ 200 mL within 30 min after administration of salbutamol 400 μg/albuterol 360 μg or equivalent dose)	48.0	33.3	0.0	86.0	FEV_1_, PEF, and rescue medication use	3
Moermans et al., 2020 [44,45]	Probiotic	NCT03341403	Phase II/III, single-centre, randomised, PCB-controlled, double-blind parallel-group study	12	46	Stable asthma treatment + Probiotical^®^ TID (containing Lactobacillus, Bifidobacterium, and Streptococcus thermophilus, 18 billion bacteria per pill)	Stable asthma treatment + PCB	PO	NA	Severe uncontrolled asthma (ACQ score > 1.5)	18.0–75.0	NA	NA	NA	ACQ and eosinophil count	3
NA, 2019 [25]	Selective BTK inhibitor	NCT03944707	Phase II, multicentre, randomised, PCB-controlled, subject- and investigator-blinded parallel-group study	12	76	BUD/FOR 160/9 μg BID + remibrutinib (LOU064) 100 mg QD	BUD/FOR 160/9 μg BID + PCB	BUD/FOR: oral inhalation; LOU064 and PCB: PO	BUD/FOR: DPI (NA)	Inadequately controlled asthma	50.7	34.2	NA	NA	FEV_1_, PEF, ACQ, rescue medication use, and symptoms control	3
NA, 2019 [27]	Pan-JAK inhibitor	NCT04150341	Phase II, multicentre, randomised, PCB-controlled, double-blind crossover study	2	24	TD-8236 150 µg, 1500 µg QD	PCB	Oral inhalation	DPI (NA)	Mild asthma with a known response to an allergen challenge (pre-bronchodilator FEV_1_ ≥ 70% predicted)	42.0	70.8	NA	NA	FEV_1_	3
Bruns et al., 2019 [46,47]	IL-4Rα inhibitor	NCT03574805	Phase I, multicentre, randomised, PCB-controlled, single-blind parallel-group study	≅1.4	42	AZD1402 (PRS-060) 2 mg, 6 mg, 20 mg, 60 mg BID	PCB	Oral inhalation	Nebuliser (InnoSpire Go)	Mild asthma (pre-bronchodilator FEV_1_ ≥ 70% predicted and FEV_1_/FVC ≥ 0.7)	28.4	88.1	0.0	NA	F_E_NO	2
NA, 2018 [23]	Anti-IL-33R mAb	NCT03393806	Phase II, single-centre, randomised, PCB-controlled, double-blind parallel-group study	12	17	Standard of care treatment + melrilimab (GSK3772847/CNTO7160) 10 mg/kg Q4W	Standard of care treatment + PCB	Melrilimab and PCB: IV	NA	Moderate to severe asthma with allergic fungal airway disease	56.9	70.6	0.0	NA	FEV_1_, ACQ, eosinophil count, F_E_NO, and AQLQ	3
NA, 2018 [40,41]	Anti-IL-33 mAb	NCT03469934	Phase II, multicentre, randomised, PCB-controlled, double-blind parallel-group study	≅18	25	Background medication with high dose ICS/LABA + single dose of etokimab (ANB-020) 300 mg/100 mL	Background medication with high dose ICS/LABA + PCB	ICS/LABA: oral inhalation; etokimab and PCB: IV	NA	Severe eosinophilic asthma	38.5	72.0	NA	NA	FEV_1_, eosinophil count, F_E_NO, and exacerbations	3
NA, 2017 [22]	anti-IL-17A mAb	NCT03299686	Phase II, multicentre, randomised, PCB-controlled, subject- and investigator-blinded parallel-group study	12	118	Standard care of treatment + CJM112 300 mg QW for 4 wks, then Q2W up to 12 wks	Standard care of treatment + PCB	CJM112 and PCB: SC	NA	Inadequately controlled moderate to severe asthma (FEV_1_ ≥ 40% and ≤90% predicted; ACQ score ≥ 1.5; total serum IgE <1 50 IU/mL; peripheral blood eosinophils < 300/μL)	56.6	39.8	NA	NA	FEV_1_ and ACQ	3
NA, 2017, [24]	Anti-IL-33R mAb	NCT03207243	Phase IIa, multicentre, randomised, PCB-controlled, double-blind parallel-group study	16	165	FP/SAL 500/50 μg BID for 2 wks, then switch to FP 500 μg BID for 2 wks, then FP dose reduction by 50% Q2W until discontinuation + melrilimab (GSK3772847/CNTO7160) 10 mg/kg Q4W	FP/SAL (500/50 μg BID) for 2 wks, then switch to FP (500 μg BID) for 2 wks, then FP dose reduction by 50% Q2W until discontinuation + PCB	FP/SAL, FP: oral inhalation; melrilimab and PCB: IV	FP/SAL, FP: DPI (Diskus)	Moderately severe asthma (bronchodilator FEV_1_ reversibility ≥ 12% and ≥200 mL; ACQ score ≥ 1.0 and <4.0)	52.9	28.5	0.0	NA	FEV_1_, PEF, ACQ, symptoms control, rescue eosinophil count, F_E_NO, exacerbations and hospitalisations, and SGRQ	3
NA, 2017 [53,54]	Anti-TSLP mAb fragment	NCT03138811	Phase I, multicentre, randomised, PCB-controlled, double-blind parallel-group study	12	28	Ecleralimab (CSJ117) 4 mg QD	PCB	Oral inhalation	DPI (PulmoSol)	Mild atopic asthma with an early and late response to a common inhaled allergen challenge	34.1	39.3	NA	NA	FEV_1_	3
NA, 2017 [26]	ENaC inhibitor	NCT03135899	Phase I, single-centre, randomised, PCB-controlled, double-blind, double-dummy crossover study	2 days	37	BI 443651 100 μg, 400 μg, 1200 μg, thrice 12 h apart	PCB	Oral inhalation	SMI (Respimat)	Mild asthma upon methacholine challenge (pre-bronchodilator FEV_1_ ≥ 70% predicted; FEV_1_ ≥ 20% reduction in response to a provocative concentration of inhaled methacholine of ≤1 mg; ACQ score < 1.5)	37.4	91.9	0.0	NA	FEV_1_	3
NA, 2017 [42,43]	MABA	NCT03378648	Phase I/II, single-centre, randomised, PCB-controlled, double-blind parallel-group study	1	48	CHF6366 40 μg, 80 μg, 160 μg, 240 μg QD	PCB	Oral inhalation	NA	Asthma (bronchodilator FEV_1_ reversibility ≥ 12% and ≥200 mL)	38.1	64.6	NA	NA	FEV_1_	3

ACQ: asthma control questionnaire; ACT: asthma control test; AHR: airway hyperresponsiveness; AQLQ: asthma quality of life questionnaire; BID: bis in die, twice daily; BTK: Bruton’s tyrosine kinase; BUD: budesonide; DP2: prostaglandin D2 receptor; DPI: dry powder inhaler; ENaC: epithelial sodium channel; FENO: fractional exhaled nitric oxide; FEV_1_: forced expiratory volume in the 1st second; FP: fluticasone propionate; FVC: forced vital capacity; ICS: inhaled corticosteroid; IL-n: interleukin-n; IL-nR: interleukin-n receptor; IV: intravenous; JAK: Janus kinase; LABA: long-acting β2 adrenoceptor agonist; mAb: monoclonal antibody; MABA: M_3_ receptor muscarinic antagonists/β_2_-adrenoceptor agonist; NA: not available; PCB: placebo; PEF: peak expiratory flow; PO: oral; QD: quaque die, once daily; Q4W: once every 4 weeks; SABA: short-acting β2 agonist; SC: subcutaneous; SGRM: selective glucocorticoid receptor modulators; SMI: soft mist inhaler; SGRQ: St. George’s Respiratory Questionnaire; TSLP: thymic stromal lymphopoietin; wks: weeks.

**Table 2:** Clinical effect of investigational agents currently evaluated in Phase I and II RCTs for the treatment of asthma compared to PCB on efficacy outcomes for which the MCID values are currently available. The investigational agents reported in this table also elicited statistically significant improvement vs. PCB (*p* < 0.05).

Outcome	Treatment	Drug Class	Delta Effect	Suggested MCID [103]	Beneficial Clinically Relevant Effect
Trough FEV_1_	Itepekimab 300 mg Q2W	Anti-IL-33 mAb	140 mL (10–270)	>100 mL	Yes
	Indacaterol maleate 150 μg QD	LABA	186 mL (129–243)	>100 mL	Yes
Peak FEV_1_	Itepekimab 300 mg Q2W + dupilumab 300 Q2W	Anti-IL-33 mAb + anti IL-4/IL-13 mAb	130 mL (10–250)	≥12% and ≥200 mL	No
PEF	Indacaterol maleate 150 μg QD	LABA	33.00 L/min (25.60–40.30)	>5.39%	?
Morning PEF	Velsecorat 720 μg QD	SGRM	16.60 L/min (8.03–25.17)	>5.39%	?
Evening PEF	Velsecorat 720 μg QD	SGRM	11.99 L/min (3.57–20.42)	>5.39%	?
ACQ	Itepekimab 300 mg Q2W	Anti-IL-33 mAb	−0.42 points (−0.73–−0.12)	>0.5 points	Borderline
	Itepekimab 300 mg Q2W + dupilumab 300 mg Q2W	Anti-IL-33 mAb + anti IL-4/IL-13 mAb	−0.32 points (−0.63–−0.01)	>0.5 points	No
	Velsecorat 720 μg QD	SGRM	−0.27 points (−0.43–−0.10)	>0.5 points	No
	CJM112 300 mg QW	IL-17A mAb	−0.23 points (−0.40–−0.06) *	>0.5 points	No
Exacerbations	Velsecorat 720 μg QD	SGRM	0.11 rate (0.04–0.25)	>−20% rate	Yes
AQLQ	Itepekimab 300 mg Q2W	Anti-IL-33 mAb	0.45 points (0.14–0.77)	>0.5 points	Borderline
	Itepekimab 300 mg Q2W + dupilumab 300 mg Q2W	Anti-IL-33 mAb + anti IL-4/IL-13 mAb	0.43 points (0.11–0.75)	>0.5 points	Borderline

* 80% Confidence Interval. ACQ: asthma control questionnaire; AQLQ: asthma quality of life questionnaire; FEV_1_: forced expiratory volume in the 1st second; IL-n: interleukin-n; LABA: long-acting β_2_-adrenoceptor agonist; mAb: monoclonal antibody; MCID: Minimal Clinical Important Difference; NA: not available; PCB: placebo; PEF: peak expiratory flow; Q2W: once every 2 weeks; QD: *quaque die*, once daily; RCT: randomised controlled trial; SGRM: selective glucocorticoid receptor modulator; TSLP: thymic stromal lymphopoietin.

## Data Availability

The data presented in this study are available in the article.
